# Simultaneous determination of five diuretic drugs using quantitative analysis of multiple components by a single marker

**DOI:** 10.1186/s13065-021-00764-z

**Published:** 2021-06-09

**Authors:** Fuchao Chen, Baoxia Fang, Peng Li, Sicen Wang

**Affiliations:** 1 Sinopharm Dongfeng General Hospital, Hubei University of Medicine, Shiyan, Hubei 442008 People’s Republic of China; 2 School of Pharmacy, Xi’an Jiaotong University, Xi’an, 710061 Shanxi People’s Republic of China

**Keywords:** Diuretic drug, Multi-components detection with a single marker, HPLC, Method development

## Abstract

**Background:**

Loop diuretics are commonly used in clinical practice to manage high fluid loads and to control fluid balance. In this paper, a novel quantitative analysis method for multiple components with a single marker (QAMS) was developed for the simultaneous determination of 5 diuretic drugs furosemide, torasemide, azosemide, etacrynic acid, and bumetanide, by HPLC. Qualitative analysis was performed using relative retention time and ultraviolet (UV) spectral similarity as the double indicator. The QAMS method was conducted with etacrynic acid as an internal reference substance. The quantities of the other four diuretics were calculated by using the relative correction factors for etacrynic acid. The quantities of the 5 diuretic drugs were also determined by the external standard method (ESM). Chromatographic separation was achieved on a Shimadzu HC-C_18_ column (150 mm × 4.6 mm, 5 µm) using 50 mM potassium dihydrogen phosphate (pH adjusted to 4.0 with phosphoric acid) with acetonitrile (64:36, v/v) as the mobile phase at a flow rate of 1.0 mL/min and a column temperature of 30 ℃.

**Results:**

Under these conditions, the 5 diuretic drugs were well separated, showing linear relationships within certain ranges. The quantitative results showed that there was no significant difference between the QAMS and ESM methods.

**Conclusions:**

Overall, the HPLC-QAMS analytical scheme established in this study is a simple, efficient, economical, and accurate method for the quantitative evaluation of 5 diuretic drugs.

## Introduction

Loop diuretics (LDs) are a kind of drug prescribed widely in clinical practices to manage the high fluid load and to control fluid balance [1]. The pharmacologic action of LDs, blocks the Na^+^-K^+^-2Cl^−^ cotransporter, which transfusions from the tubular lumen into tubular cells. They inhibit Na^+^ and Cl^−^ reabsorption in the thick ascending limb of the loop of Henle and cause increased secretion of water, K^+^, Na^+^, and Cl^−^ [2]. Furosemide, torasemide, azosemide, bumetanide, and etacrynic acid are examples of this class of diuretics [3].

**Fig. 1:**
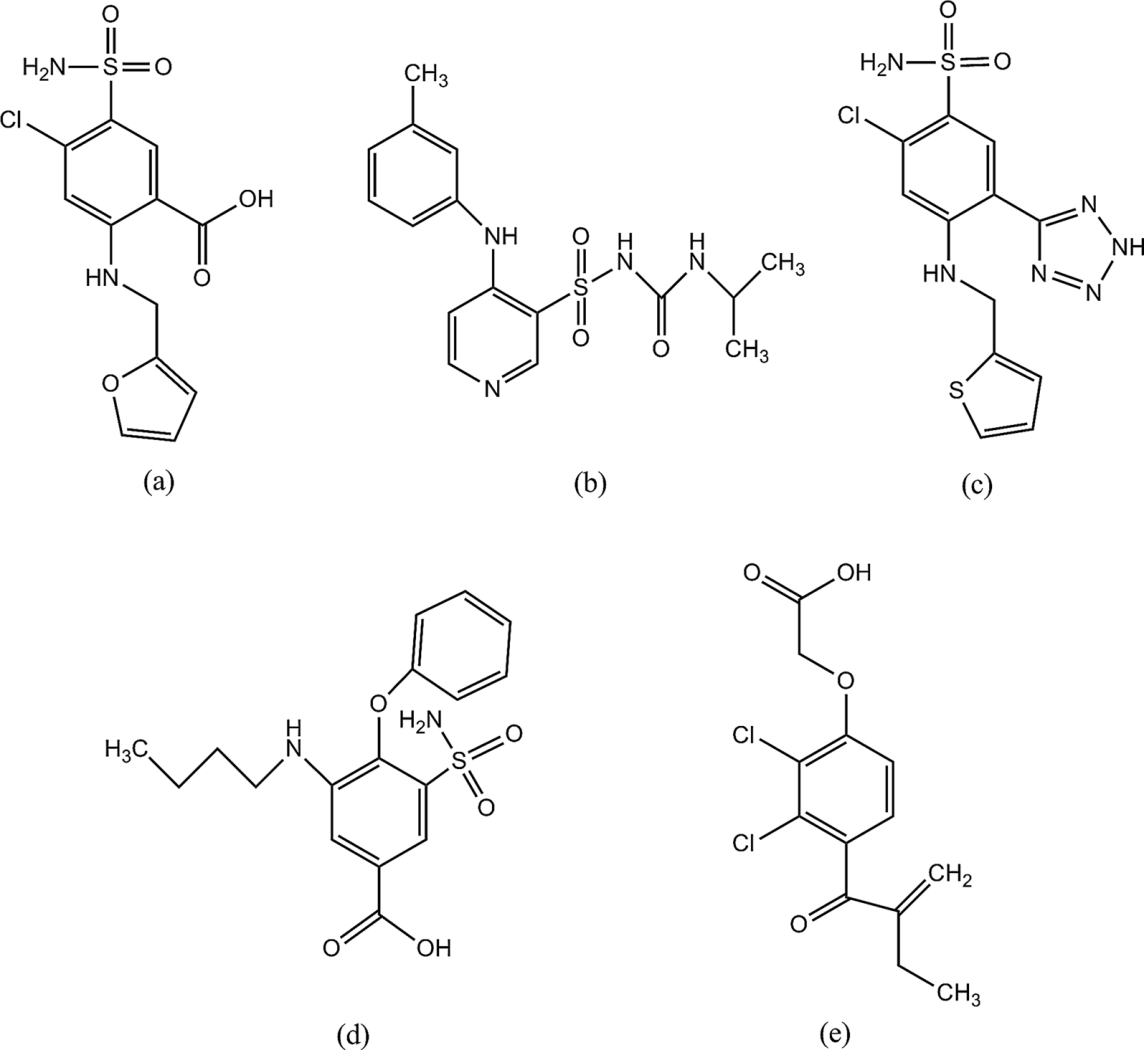
Chemical structures of furosemide (**a**), torasemide (**b**), azosemide (**c**), bumetanide (**d**) and etacrynic acid (**e**)

**Fig. 2:**
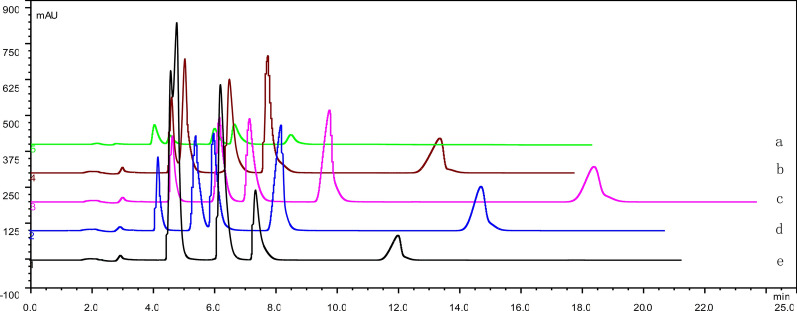
A typical chromatogram of standard drug mixture samples. Chromatographic conditions were acetonitrile-0.05 mol·L^−1^ potassium dihydrogen phosphate (36: 64, v/v); the buffer pH was varied. a. pH 3.0; b. pH 3.5; c. pH 4.0; d. pH 4.5; e. pH 5.0

Furosemide (Fig. 1a), a 5-sulfamoylbenzoic acid derivative LD, has been the most widely prescribed with regard to administration via continuous infusion, and the chemical name is 5-(aminosulfonyl)-4-chloro-2-([2-furanylmethyl]amino)benzoic acid [4]. Torasemide (Fig. 1b), a nonacidic LD, has a long half-life, long time of action, and higher bioavailability than the other LDs, and its chemical name is 1-[4-(3-methylphenyl)aminopyridine-3-based]sulfonyl-3-isopropylurea [5]. Azosemide (Fig. 1c), [2-chloro-5-(1H-tetrazole-5-yl)-N4-(2-thenyl) sulfanilamide], is a sulfonamide LD, that is used clinically in the treatment of oedema of various geneses and hypertension [6]. Bumetanide (Fig. 1d), a 5-sulfamoylbenzoic acid derivative LD, has the chemical name 3-(aminosulfonyl)-5-(butylamino)-4- phenoxybenzoic acid [7]. Etacrynic acid (Fig. 1e), [2,3-dichloro-4-(2-methyl- enebutyryl)phenoxy)-acetic acid], is chemically different from all other known diuretic agents, and is now mainly used for patients with severe sulfonamide allergies that prohibit the use of other LDs [8].

Currently, several analytical methods have been reported by HPLC for the quantification of furosemide, torasemide, azosemide, bumetanide, or etacrynic acid either alone or in combination with other drugs, both in their prescribed forms and in biological fluids [9–21]. The above mentioned methods, however, have not been employed for the simultaneous quantification of the five LDs in any pharmaceutical formulation. The QAMS method is able to simultaneously determine the concentrations of multiple components by a single reference standard, which greatly reduces the cost and reduction of the analysis time of the experiment [22–29]. Thus, the present study developed a rapid HPLC-QAMS method for the determination of the five LDs. The developed QAMS method was evaluated by comparing the calculated results with an external standard method (ESM). This method was then successfully applied for the quality evaluation of formulas of the five LDs in hospitals.

## Experimental

### Materials and reagents

Five reference substances (furosemide, torasemide, azosemide, bumetanide, and etacrynic acid) were obtained from the National Institutes for Food and Drug Control (Beijing, China). The purity of each of the five reference substances was higher than 99%. Chromatographic grade methanol and acetonitrile were purchased from Kemiou Chemical Reagent Co., Ltd. (Tianjin, China). AR grade of potassium dihydrogen phosphate (KH_2_PO_4_), triethylamine, and phosphoric acid were obtained from Sinopharm Chemical, Shanghai, China. Pharmaceutical formulations containing furosemide, torasemide, azosemide, bumetanide, or etacrynic acid were obtained commercially.

### Apparatus and analytical conditions

Chromatographic separations were achieved on an Agilent HPLC 1260 system equipped with a diode array detector (G4212B), autosampler (G1329B), quaternary pump (G1311C), and column oven (G1316A). A digital workstation with ChemStation software Version C.01.10 served as both a controller and data manager for the overall system. A Shimadzu HC-C_18_ column (150 mm × 4.6 mm, 5 µm) was applied during the study under the following analytical conditions: the mobile phase was composed of acetonitrile with 0.05 mol/L KH_2_PO_4_ (pH adjusted to 4.0 using diluted phosphoric acid) (36: 64, v/v) at detection wavelengths of 278 nm, at a flow rate of 1.0 mL/min. The column temperature was retained at 30 °C, and a sample volume of 20 µL was injected by an automatic sampler.

### Laboratory prepared mixture standard solution

Furosemide, torasemide, azosemide, bumetanide, and etacrynic acid references were weighed precisely to 25.0 mg each, and then the compounds were dissolved in the mobile phase in a 50 mL volumetric flasks. These stock standard solutions were stored at 4 °C and warmed to room temperature before use. Mixed working solutions of the five LDs were prepared, and the mobile phase was added to form a mixed reference solution with a concentration of approximately 50 µg/mL of the reference substance.

### Method validation

The method was validated according to the International Conference of Harmonization (ICH) guidelines [30]. The following parameters were investigated: linearity, precision, stability, accuracy, limit of detection and robustness.

### Linearity and range

The linearity and range of the method were evaluated with the standard solution of the five LDs at six different concentrations. The concentrations ranged from 2.5 to 150.0 µg/mL for the five LDs, respectively. The mixed test working standard solutions were prepared by appropriate dilution of the stock solutions with mobile phase to the required concentrations for plotting the calibration curves. A 20 μL aliquot of each working solution was injected in triplicate into the chromatographic system (n = 3). The standard curves of the five LDs were constructed from the different concentrations of the mixed solution. Chromatograms were recorded, and the standard calibration curve was generated with peak area as the Y-axis (Y) and the concentration (µg/mL) of each standard solution as the X-axis (X).

### Precision

The mixed standard solution at the same concentration was continuously injected six times on the same day or on different days according to the chromatographic conditions described in Section analytical conditions. The relative standard deviation (RSD) values were evaluated by the chromatographic peak area of the five LDs.

### Stability

The same mixed working standard solutions were injected into the HPLC at 0, 1, 2, 4, 6 and 8 h with the same mobile phase. The RSDs of the five peaks areas were determined.

### Recovery

The accuracy of the assay method was calculated in triplicate by adding known amounts of the five LD reference substances to commercial sample solutions. Each solution was prepared at three concentrations (i.e., 40, 50, and 60 µg/mL), and each solution was injected into HPLC in triplicate. Then, the peak areas were recorded, and the average recovery, and RSD % of each LD were calculated.

### Robustness

The robustness study was conducted by examining the samples on three different HPLC instruments: a Dionex U3000, an Agilent 1260 series, and a Shimadzu LC-20A. Three different models of chromatographic columns were used, including an Agilent Zorbax Extend C_18_, Shimadzu HC-C_18_ column, and Kromasil C_18_ (150 mm × 4.6 mm, 5 um). The samples were determined to have various pH values (3.8, 4.0, and 4.2), flow rates (0.98, 1.0, and 1.02 mL/min), flow volume (34: 66, 36: 64, and 38: 62) of the mobile phase, and column temperatures (29, 30, and 31 °C). The separation degree and % RSD of the five LDs were investigated.

### Qualitative investigation

#### UV spectral similarity

The mixed standard solutions of five LDs were injected into HPLC under the above analytical conditions. The chromatogram maps and UV spectra were recorded, and the similarity of the original and first-order spectra of the five LDs was analyzed by the ChemStation software Version C.01.10.

#### Relative retention time

Under the analytical conditions described in sections analytical conditions and robustness, the same standard solutions of the five LD mixtures were tested and peak retention times were recorded to determine the relative retention time (RRT). The RRT is calculated by the following formulae (1):

1 $$ {\text{RRT }} = ({\text{t}}_{{\text{A}}} - {\text{t}}_{0} ) \, /({\text{t}}_{{\text{R}}} - {\text{t}}_{0} ) $$

where t_0,_ t_R_ and t_A_ represent the retention times of urine pyrimidine, etacrynic acid, and analyte, respectively.

### Quantitative analysis of multiple components by a single marker (QAMS)

The application of the QAMS method in the quality control of the five diuretic drugs was based on the relative correction factor (RCF) of each component, which is proportional to the detection signal in a certain concentration range. In this study, we selected etacrynic acid as the internal standard, and used Eq. (2) to calculate the RCF of the other diuretics.

2 $$ {\text{RCF}} = \frac{{{\text{RCF}}_{{\text{s}}} }}{{{\text{RCF}}_{{\text{i}}} }} = \frac{{{\text{A}}_{{\text{s}}} /{\text{C}}_{{\text{s}}} }}{{{\text{A}}_{{\text{i}}} /{\text{C}}_{{\text{i}}} }} $$

where *A*_*s*_ is the peak area of the internal standard (etacrynic acid), *C*_*s*_ is the concentration of the internal standard (etacrynic acid), *A*_*i*_ is the peak area of other investigated components i, and *C*_*i*_ is the concentration of other investigated component i in the sample solution.

From the Eq. (2), we can derive the Eq. (3)

3 $$ C_{i} = RCF \times C_{s} \times \frac{{A_{i} }}{{A_{s} }} $$

We can use Eq. (3) to calculate the concentration of each component of the sample solution. Additionally, the effects of the different HPLC systems, the pH of the mobile phase, the gradient elution scheme, the flow rate, the injection volume and the column temperature given in section robustness for the RCF were investigated.

### Analysis of the five LDs in commercial injections and tablets

To determine the content of furosemide, bumetanide, and torasemide, commercially available injections of the 3 diuretics were prepared with the mobile phase. To determine the content of azosemide and etacrynic acid in conventional tablets, ten tablets were weighed, and disintegrated by shaking for 1 min with 10 mL water in a 100 mL volumetric flask. 40 mL acetonitrile was added. The samples were ultrasonically treated for 20 min, and diluted with purified water to a volume of 100 mL. Then, the samples of the 5 diuretics were injected into the HPLC according to the above described analytical method.

## Results and discussion

### Optimization of analytical conditions

**Fig. 3:**
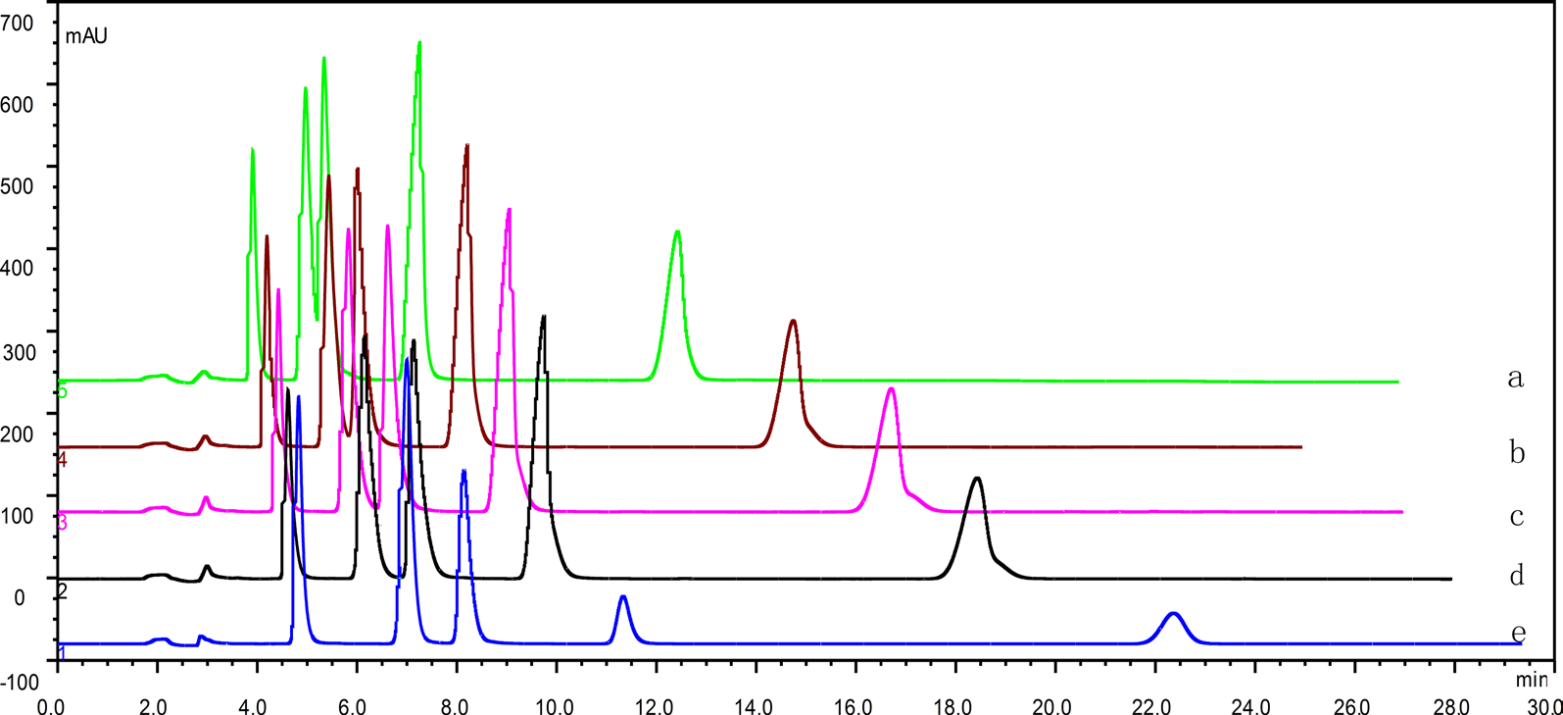
A typical chromatogram of standard drug mixture samples. Chromatographic conditions were acetonitrile-0.05 mol·L^−1^ potassium dihydrogen phosphate (phosphoric acid to adjust pH to 4.0); the acetonitrile content was varied. a. acetonitrile content 30; b. acetonitrile content 34; c. acetonitrile content 36; d. acetonitrile content 38; e. acetonitrile content 40

To establish a method for the simultaneous determination of five LDs by HPLC with DAD detection, solutions of methanol/water, methanol/water containing triethylamine or phosphoric acid, acetonitrile/water, and acetonitrile/water containing phosphoric acid or triethylamine were first used to separate the compounds, but the selectivity between these five LDs was not optimal. Then, we chose a 0.05 mol/L solution of potassium dihydrogen phosphate with acetonitrile, which provided a different selectivity than the other conditions and thus a better separation between the five LDs. Representative HPLC chromatograms for the simultaneous separation of the five LDs with different buffer pH or acetonitrile contents are shown in Fig. 2 and Fig. 3. The mobile phase composed of acetonitrile—0.05 mol/L of KH_2_PO_4_ (pH adjusted to 4.0 using diluted phosphoric acid) (36: 64, v/v) provided a short analysis time that did not exceed 25 min while maintaining acceptable resolution between compounds. Under the above conditions, the retention time for torasemide, furosemide, azosemide, etacrynic acid, and bumetanide were observed at 4.8, 6.3, 7.6, 9.7 and 18.5 min, respectively.

### Method validation

The linear range, precision, stability, and recovery results are showed in Tables 1 and 2. The linear range of all five LDs was wide and showed a good linear relationship (r > 0.999). As shown, the precision was measured in two parts: intraday precision (with RSD values between 0.1 and 1.5%), and interday precision (with RSD values between 0.4 and 2.3%), which showed that the instrument had good precision. The RSD% of the peak areas for furosemide, torasemide, azosemide, bumetanide, and etacrynic acid for stability were 1.1, 0.7, 1.3, 1.1, and 0.5%, indicating that the sample solutions were stable over 8 h. The recovery results revealed average recovery rates from 97.2 to 102.1% with an RSD < 1.9%, indicating that the method had good accuracy. All of the above results show that the established methods were effective and reliable.

**Table 1:** Regression equation, correlation coefficient (r), linear range and detection limit of the five diuretic drugs

Studied drug	Regression Equation	r	(Linear Range)/ (μg·mL^−1^)	(Detection Limit)/μg·mL^−1^
Etacrynic acid	y = 20.301x − 1.624	0.9993	2.5–150	0.25
Torasemide	y = 33.204x + 3.131	0.9998	2.5–150	0.08
Furosemide	y = 27.634x − 0.970	0.9999	2.5–150	0.05
Azosemide	y = 43.381x + 8.666	0.9992	2.5–150	0.13
Bumetanide	y = 36.124x + 1.320	0.9999	2.5–150	0.20

**Table 2:** Recovery and precision test of the developed HPLC analysis method

Studied drug	Measured concentrations (μg/mL)	Accuracy %	Precision RSD (%)	
			Intra-day	Inter-day
Etacrynic acid	40.0	98.3	0.9	1.5
	50.0	97.7	0.5	0.4
	60.0	100.4	0.1	0.5
Torasemide	40.0	96.7	0.4	0.7
	50.0	99.1	0.3	0.8
	60.0	99.6	1.5	0.9
Furosemide	40.0	100.6	0.5	1.3
	50.0	102.1	0.2	1.2
	60.0	98.3	1.2	2.3
Azosemide	40.0	97.2	0.6	1.5
	50.0	98.2	0.3	0.6
	60.0	98.4	0.9	0.5
Bumetanide	40.0	97.5	1.3	1.7
	50.0	100.8	0.8	1.9
	60.0	99.6	0.7	1.4

### Qualitative investigation

#### UV spectral similarity

**Fig. 4:**
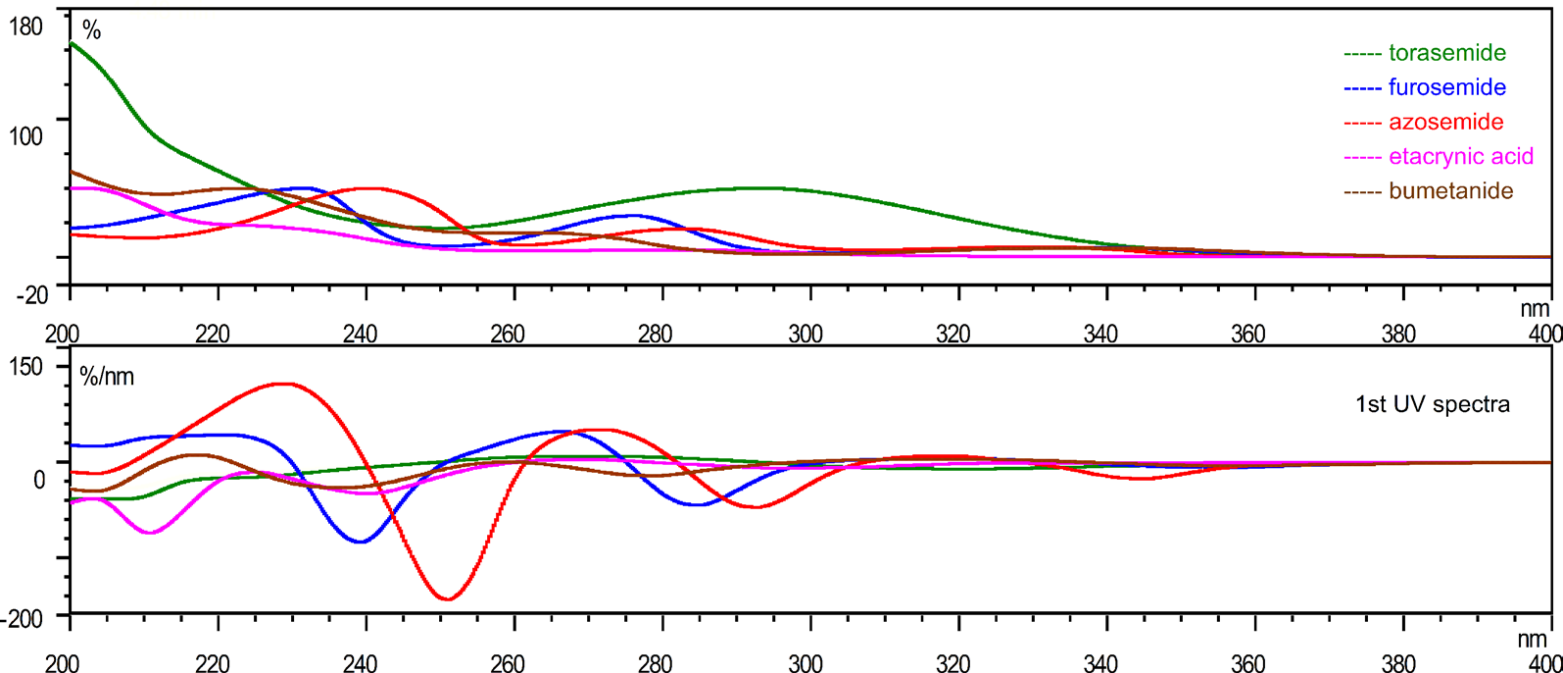
UV spectra and 1st UV spectra of furosemide, torasemide, azosemide, bumetanide and etacrynic acid

The similarity of the UV spectra is due to the chemical structure of compounds, and different compounds have different UV absorption spectra, which can be used to characterize the LD compounds. The original and first-order spectra, illustrating the similarities of the five diuretics are shown in Table 3–4, and the vector graph of the five diuretics is shown in Fig. 4, which indicates that the first-order spectra after derivation show more characteristic details than the original spectra. The similarity of the UV spectra can be used for the qualitative identification of the five diuretics, which improves the accuracy and other characterization.

**Table 3:** UV spectra similarity of the five diuretic drugs

Studied drug	Torasemide	Furosemide	Azosemide	Etacrynic acid	Bumetanide
Torasemide	1.0000				
Furosemide	0.7494	1.0000			
Azosemide	0.6574	0.8550	1.0000		
Etacrynic acid	0.9219	0.7761	0.6553	1.0000	
Bumetanide	0.8965	0.8499	0.7544	0.9451	1.0000

**Table 4:** 1st UV spectra similarity of the five diuretic drugs

Studied drug	Torasemide	Furosemide	Azosemide	Etacrynic acid	Bumetanide
Torasemide	1.0000				
Furosemide	− 0.1078	1.0000			
Azosemide	− 0.1712	0.1698	1.0000		
Etacrynic acid	0.8132	0.0873	− 0.0475	1.0000	
Bumetanide	0.3288	0.3369	0.0457	0.1218	1.0000

#### Relative retention time

The HPLC methods used in the literature mostly use the retention time to carry out the qualitative analysis. In multicomponent qualitative analysis, heavy use of the reference substance increases the detection cost; in the detection process, the reference substance often cannot be rapidly obtained, and the retention time is affected by the instrument, brand of chromatographic column, column temperatures, and flow rates or pH of the mobile phase. In addition, the five LD compounds with similar chemical structures and physical properties are difficult to qualitatively investigate base on the retention time of the components. In this experiment, the RRTs were chose as a qualitative analysis index, and their robustness was investigated. The robustness results of the RRTs for the five LDs are shown in Table 5. The results showed that the RRTs were not affected by different instruments, flow velocity, mobile phase volume, pH value or column temperature (RSD was less than 5.0%). In the same system, different HPLC columns gave RRTs with a RSD > 31.0%, which failed to satisfy the requirements of qualitative analysis. The differences between RRTs can mainly be attributed to different manufacturers, and are associated with the properties and preparation of packing materials. To this end, we herein restricted the conditional parameters and used spectral similarity and RRT as the double indicator to qualify the qualitative analysis.

**Table 5:** Effects of different instruments, columns, column temperatures, flow rates, volume and pH on RRTs (n = 3)

Effects Factor	Torasemide	Furosemide	Azosemide	Etacrynic acid	Bumetanide
RSD%					
Chromatographic Columns	38.95	36.77	35.97	-	31.67
HPLC Instruments	1.09	1.24	3.38	-	2.34
Column Temperatures	1.58	1.36	0.54	-	0.93
Flow Volume of Mobile Phase	2.10	2.11	0.93	-	1.22
Flow Rate of Mobile Phase	1.22	0.75	0.40	-	0.005
pH Value of Mobile Phase	4.87	3.21	3.99	-	3.71
$\bar \chi$	0.31	0.49	0.63	-	2.11
S	0.001	0.001	0.001	-	0.009
$\bar \chi \pm 3{\rm{S}}$	0.31 ± 0.003	0.49 ± 0.004	0.63 ± 0.002	-	2.11 ± 0.028

### Quantitative study of QAMS

Inspired by the HPLC-based ESM analytical method, QAMS uses a single component as the internal standard to simultaneously quantitatively measure itself and other analytes by calculating relative correction factors (RCFs). In recent decades, the QAMS method has been widely used in the evaluation of a large number of Chinese herbal medicines [22–28], drugs [29] and drug impurities [31, 32]. In this study, etacrynic acid was chosen as an internal reference to calculate the RCF of the other four diuretics. To evaluate the robustness of the RCF, the influence of the pH values of mobile phase, ratio and flow rates of the mobile phase, chromatographic column types, chromatographic column temperatures, and injection volumes were investigated. The RCFs of the five LDs are shown in Table 6. The results show that the RCF experiment is repeatable under different analytical conditions, which ensures that the QAMS method can be well applied to routine analysis. The proposed HPLC-QAMS and HPLC-ESM method is very simple, cost-effective, and accurate as it requires simple reference materials, reagents and chemicals only. Previous reported HPLC researches in this area has reported retention times that were one to three times longer than those reported in this study, which demonstrates the efficiency of this method [17, 18]. The clean separation of peaks also proves that the method is capable of differentiation between the different diuretic drugs and for individual quantification.

**Table 6:** Relative correction factor of the five diuretic drugs

Studied drug	Wavelength (nm)	RSD/%	Relative correction factor
Etacrynic acid	277	-	1
Torasemide	292	0.18	0.3232
Furosemide	334	0.35	0.9024
Azosemide	326	0.63	0.8064
Bumetanide	260	0.67	0.5566

### Sample analysis

The quantities of the five LD samples determined by HPLC-ESM and HPLC- QAMS are shown in Table 7. Comparative analysis indicated that there was no significant difference between HPLC-ESM and HPLC-QAMS (RSD values were < 3.0%), which shows that the above method is accurate and reliable.

**Table 7:** Results of the comparison of the five diuretic drugs

Studied drug	The QAMS methods		The ESM methods	
	Accuracy (%)	RSD (%)	Accuracy (%)	RSD (%)
Etacrynic acid tablet	95.1	2.1	96.2	1.9
Torasemide injection	97.4	1.3	96.8	1.4
Furosemide injection	103.2	0.7	102.9	1.0
Azosemide tablet	98.5	1.8	99.1	2.2
Bumetanide injection	102.4	1.6	101.6	1.4

## Conclusion

In this paper, a method for the simultaneous determination of five diuretic drugs by HPLC-ESM and HPLC-QAMS was developed. In the HPLC-QAMS process, etacrynic acid was used as an internal reference to calculate the relative correction factor of the other four diuretics, and the influencing factors such as different chromatographic columns, instruments, column temperatures, mobile phases and flow velocities were investigated. The above established method was successfully applied to the qualitative identification and quantitative analysis of five LDs. Because the QAMS analysis method can provide reliable results, save reference materials and shorten analysis time, it has great potential for an enhanced role in hospital-based quality control and qualitative identification and quantitative analysis of diuretics in other medicinal materials.

## Data Availability

The majority of the data used to support the findings of this study are included within the article. Other data are available from the corresponding author upon request.

## Abbreviations

LDs: Loop diuretics
HPLC: High performance liquid chromatography
QAMS: Quantitative analysis of multiple components with a single marker
ESM: External standard method
RSD: Relative standard deviation
RCFs: Relative correction factors
